# A cross-sectional evaluation of binge-eating behavior and its correlation with anxiety disorders among adolescents in Northern Saudi Arabia: Implications for future generations

**DOI:** 10.1192/j.eurpsy.2025.1008

**Published:** 2025-08-26

**Authors:** A. M. Alhuwaydi

**Affiliations:** 1 internal medicine, jouf university, sakaka, Saudi Arabia

## Abstract

**Introduction:**

Binge-eating behavior and anxiety disorders pose a significant public health issue worldwide, as it has severe implications for both the physical and mental health of the adolescent population. The present study evaluated the prevalence of binge-eating behavior, anxiety disorders, and associated factors among the northern Saudi adolescent population.

**Objectives:**

assessed the correlation between binge eating and anxiety.

**Methods:**

The present population-based cross-sectional study was carried out among adolescents in the Aljouf region of Saudi Arabia from June 2023 to December 2023. A total of 384 eligible participants were selected using the convenience sampling method. The present study used a pretested Arabic version of the binge eating scale (BES) and Hamilton Anxiety Scale (HAM-A) to assess the binge-eating behavior and anxiety disorders among the target population. The Spearman correlation test determined the strength and direction of the correlation between BES and HAM-A scores. Furthermore, logistic regression analysis was applied to find the associated factors for binge-eating behavior among the study participants.

**Results:**

Of the 384 participants, moderate and severe binge-eating behaviors were found among 11.2% and 8.3% of the respondents, respectively. Regarding the severity of anxiety as assessed by the HAM-A scale, mild, moderate, and severe anxiety were shown among 12.8%, 9.6%, and 7.5% of the participants, respectively. Also, the study found a positive correlation between binge eating and anxiety scores, with a correlation coefficient of 0.26 and a p-value of 0.001. Furthermore, being female (p = 0.001), moderate (p = 0.004), and severe anxiety (P = 0.001) were significantly associated with binge-eating behavior.

**Conclusions:**

The present research findings advocate for the implementation of targeted interventions and support services aimed at decreasing binge-eating behavior and anxiety, thereby promoting the overall well-being of adolescents and building stronger future generations. Moreover, it is recommended that optional courses about binge eating be incorporated into the curricula of schools and universities.

**Disclosure of Interest:**

None Declared

